# Mechanical Behavior of Hardened Printed Concrete and the Effect of Cold Joints: An Experimental Investigation

**DOI:** 10.3390/ma17246304

**Published:** 2024-12-23

**Authors:** Theresa Glotz, Inken Jette Rasehorn, Yuri Petryna

**Affiliations:** 1 Chair of Structural Mechanics, Technische Universität Berlin, Gustav-Meyer-Allee 25, 13355 Berlin, Germany; 2 Chair of Building Materials and Construction Chemistry, Technische Universität Berlin, Gustav-Meyer-Allee 25, 13355 Berlin, Germany; i.rasehorn@tu-berlin.de

**Keywords:** 3D concrete printing (3DCP), additive manufacturing, hardened concrete, mechanical properties, cold joint, anisotropy, experiment, digital image correlation (DIC)

## Abstract

The adaptation of 3D printing techniques within the construction industry has opened new possibilities for designing and constructing cementitious materials efficiently and flexibly. The layered nature of extrusion-based concrete printing introduces challenges, such as interlayer weaknesses, that compromise structural integrity and mechanical performance. This experimental study investigates the influence of interlayer orientation and the presence of cold joints (CJ) on mechanical properties, such as stiffness and strength. Three-point bending tests (3PBT) and optical measurement techniques are employed to correlate these properties with the structural response of hardened printed concrete. The analysis determines key properties like Young’s modulus and flexural tensile strength and evaluates them statistically. The investigation examines crack development and failure mechanisms, relating them to the material properties. The findings reveal a strong dependency of material properties and crack formation on layer orientation. Specimens with interlayers aligned parallel to the loading direction exhibit significantly inferior mechanical properties compared with other orientations. The presence of CJ considerably influences the progression of crack formation. This research contributes to a deeper understanding of the structural performance of printed concrete.

## 1. Introduction

In response to the growing demand for sustainable and efficient building methods, 3D concrete printing (3DCP) has emerged as an innovative approach within the construction sector since the mid-1990s [1]. The combination of digital design and additive manufacturing in 3DCP provides key benefits such as design flexibility, optimized material usage, increased time efficiency, enhanced safety standards, and reduced costs [1,2,3]. Together, these factors contribute to the sustainability of construction processes.

Additive manufacturing in construction encompasses several techniques, including particle-bed binding and powder bed fusion [2,4]. However, extrusion-based 3DCP stands out due to its unique challenges, requiring detailed investigation across several critical aspects. The fresh material must satisfy essential criteria for pumpability, extrudability, and buildability [5,6,7]. Consequently, developing advanced concrete mixtures that meet these requirements is fundamental to achieving a printable and durable mixture. Enhancing the structural performance of printed elements also requires significant research on the integration of reinforcement [3]. Additionally, other key research areas focus on digital developments, such as design optimization, process control, and computational modeling [8,9].

While all these aspects play an important role in advancing the field, the hardened properties are crucial for ensuring structural integrity. In contrast to conventionally cast concrete, the layer-by-layer deposition process leads to anisotropic material behavior, which significantly affects the mechanical performance of printed concrete [10].

The anisotropy is evident in the directional dependency of material properties due to different relations between load application and layer orientation. In an experimental and numerical study, Kumar et al. reported on the influence of interlayer orientation and loading direction on the compressive and flexural strength [11]. Similar influences on compressive and flexural strength were observed for a highly thixotropic printed concrete [12], as well as on tensile splitting strength for a mix containing recycled sand [13]. Other studies further confirmed this orientation dependency [14,15,16,17].

Various factors contribute to the effect of directional dependency in printed concrete. Ding et al. provide a critical review of recent advances in research on interlayers in 3DCP. From a microscopic perspective, factors such as moisture transport processes and pore structure distribution play significant roles and influence each other [18]. Higher local porosity has been confirmed through air void content analysis [15,19] and the presence of macropores [15,20]. Additionally, Keita et al. identified a reduced interlayer bond strength resulting from water evaporation from the exposed surface during the interval between layer deposition [21].

The interval time between layer depositions is a decisive factor. As the interlayer interval time in the deposition of successive layers increases, weak interfaces can form due to inadequate bonding. These cold joints (CJ) have been investigated in multiple studies. Wolfs et al. found that the bond strength is influenced by the interlayer interval time, observing effects in bending tests for intervals up to 24 h [22]. Similarly, Meurer and Classen [23] reported on decreasing interface properties in three-point bending tests (3PBT) for intervals up to 40 min, though compressive load performance was less affected. Conversely, influences not only on interlayer bond strength but also on compressive strength were observed in [24] for defined intervals up to 60 min. Sanjayan et al. further correlated the interlayer interval time dependency to the surface moisture content at the interlayer interfaces, concluding that a dry surface prevents bond development [14]. Using scanning electron microscopy, Nerella et al. analyzed interface quality for varying interlayer intervals and compressional and flexural testing after 1 and 28 days and found the formation of large cavities at the interface [15]. A dependence of the interlayer adhesion was also found for the vertical position of the interlayer, with lower bond strength for interfaces located at the bottom due to elongated and flat pores in the corresponding interlayer zone [25]. Other studies focused on mixtures with coarse aggregate and CT scans of pore size and distribution, which correlated with reduced direct tensile strength as interval time increased [26]. The same trend was observed in [27] for splitting prism tests. Additionally, Thakur et al. identified a correlation between interlayer bond deterioration and fracture behavior on 3PBT with notched beams [28]. Influences of printing intervals were also studied regarding the interfacial shear behavior in [29], finding a decreasing interfacial shear strength for increasing interlayer interval times and for higher ratios of recycled sand. Pan et al. proposed a parameter, the maximal operational time (MOT), to define the maximum allowable interval time for preserving bond quality [30].

Several approaches have been proposed to address the challenges associated with interlayer interval time. Nerella et al. improved bond performance by optimizing the mixture design, achieving better results with supplementary cementitious materials compared with a pure Portland cement binder [15]. Marchment et al. increased bond strength by applying a cementitious paste at the interface [31]. Adjusting printing parameters, such as printing speed and nozzle standoff distance, resulted in improved bond strength for a test series with geopolymer mortar at lower values of these parameters [32]. Furthermore, Wu et al. obtained promising results by incorporating superabsorbent polymers in mixtures with recycled sand in order to counter moisture loss and support interlayer CSH production [33].

The field of research on hardened properties and influencing factors in 3DCP is broad and variable, often influenced by differences in mixtures, test setups, and methodologies. Many researchers have highlighted the need for standardized specimen preparation and consistent testing methods to assess the mechanical properties of 3DCP [18,23,34]. In response, the RILEM TC 304-ADC established an interlaboratory study to standardize mechanical property assessment for additively manufactured cementitious materials, aiming to create comparability across studies [35]. Nevertheless, understanding the mechanical parameters of printed concrete within the broader context of its structural mechanical behavior is essential for interpreting results accurately. Babafemi et al. advise investigating the mechanics underlying weak bond strength [36], while Zhang et al. note the need for research that addresses both the targeted hardened properties and the anisotropy induced by the production process of 3DCP [7].

To contribute to these research needs, the present study investigates two series of hardened printed concrete specimens with different layer orientations in 3PBT, manufactured according to the specifications in [35]. The test series includes specimens with and without CJ to examine the effect of interlayer interval time on the mechanical response. Advanced characterization through digital image correlation (DIC) is used for precise optical measurements, consistent with applications in recent studies [13,37,38,39,40]. The use of DIC enables the correlation of mechanical properties with the overall structural response, a relationship that remains underexplored in existing studies. Failure mechanisms are evaluated with respect to interlayer dependency at a macroscopic scale, excluding additional variables such as reinforcement. This study aims to provide insights into key aspects of hardened printed concrete behavior, particularly anisotropy and the impact of CJ, to advance understanding of its structural performance.

## 2. Materials and Methods

### 2.1. Printing Conditions and Concrete Mix Design

**Figure 1:**
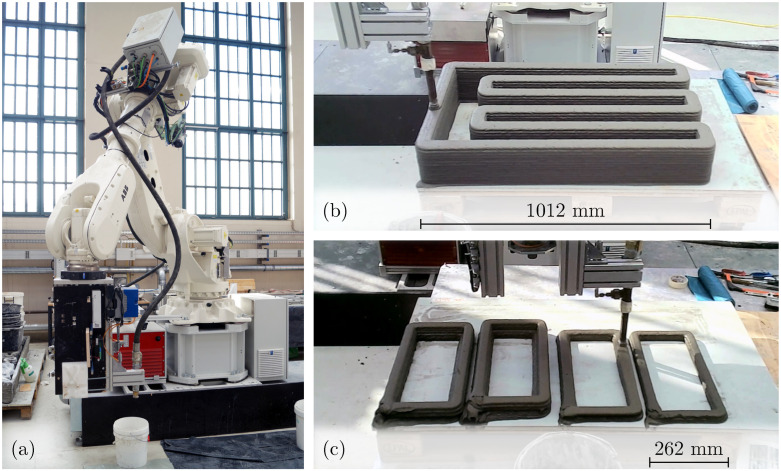
(**a**) ABB Ltd IRB 6700 robotic arm of the printing facility at Technische Universität Berlin used to manufacture the printed specimens. (**b**) Printing process for producing the object for specimen extraction without cold joints (CJ). (**c**) Printing process after a 90 min pause, resulting in objects containing CJ.

The printing process is conducted using a 6-axis ABB Ltd IRB 6700 robotic arm (Mannheim, Germany; Figure 1a) with a supplying batch system consisting of a Beckschulte P20 pump and mixer (Siegburg, Germany) and a total hose length of 15 m. The print head, equipped with an inner nozzle diameter of 25 mm, operates at a printing speed of 45 mm $s^{-1}$ and a flow rate of 1.24 ${\mathrm{dm}}^{3}$/min, resulting in a filament width of 52 mm and height of 10 mm. The material used for printing is a cement-based mortar mainly consisting of CEM I 52.5 R that is chosen due to the quick setting after extrusion, sand with a particle size of 0 mm to 1 mm, limestone powder (LS), fly ash (FA), microsilica (MS), basalt fibers of 6 mm length, tap water, superplasticizer (SP) ViscoCrete-2620, and a deaerator agent PerFin. The precise composition of the mixture is provided in Table 1.

Two printing configurations are being realized. In the first configuration, consecutive layers are deposited continuously with a printing time of 78 s per layer (see Figure 1b). In the second, printing is paused for 90 min upon reaching half the intended specimen height, after which the remaining layers are added with a printing time of 34 s per layer (Figure 1c). This process results in CJs at the mid-height of the printed object. The printed objects are cured under foil for 24 h (20 °C ± 2 °C and 40% ± 5% relative humidity) before being moved into climate-controlled conditions (20 °C ± 2 °C and 50% ± 12% relative humidity).

### 2.2. Test Series

The test series follows the guidelines of the RILEM TC 304-ADC interlaboratory study on the mechanical properties of additively manufactured cementitious materials [35]. However, the results presented in this paper are not part of the study. The focus of this work lies on a detailed mechanical analysis and therefore requires modifications to certain testing conditions like the load application.

**Figure 2:**
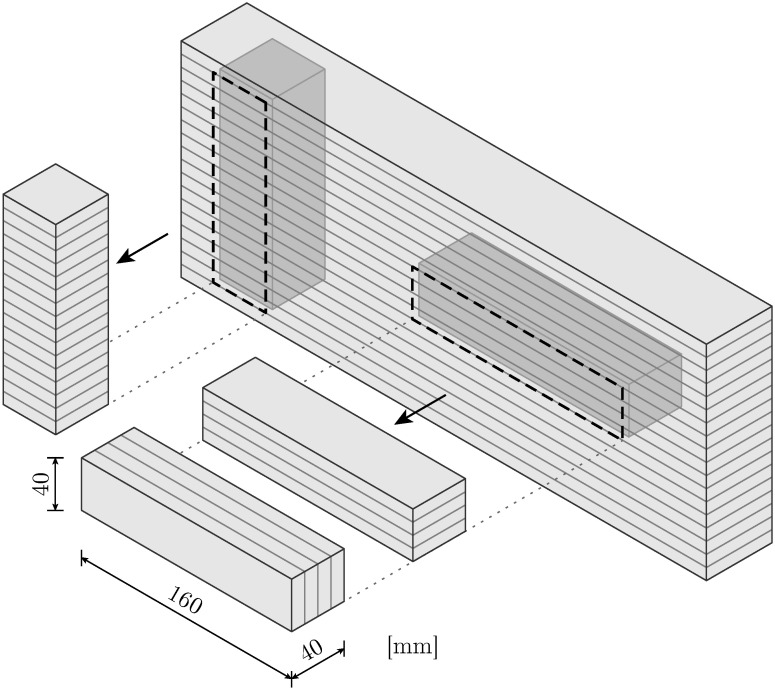
Schematic overview of the extraction of specimens from the printed object.

Three different combinations of interlayer and load orientation are realized to systematically study the anisotropic characteristics of the printed material. The specimens are extracted from the straight wall of a printed object by wet sawing on each outer surface. Horizontal extraction is performed to obtain specimens with interlayers oriented both horizontally and lengthwise vertically by rotating them by 90° (see Figure 2). This extraction method is equally applied for specimens containing CJ, where the CJ lie in the middle plane. Additionally, upright extraction is applied to printed objects without CJ to generate specimens with interlayers aligned along their longitudinal axis; see Figure 2. The height of the printed object with CJ limits extraction to horizontal orientation. According to the normative testing standards for cement-based mortars, all specimens are prepared to the target dimensions of 40 × 40 × 160 mm.

### 2.3. Experimental Setup

**Figure 3:**
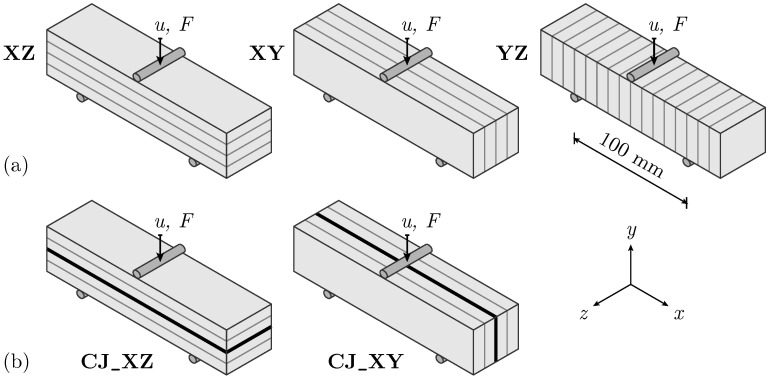
Overview of experimental test setup: Displacement-controlled three-point bending test (3PBT) (**a**) with layers oriented in XZ-, XY-, and YZ-directions, (**b**) with CJ and layers oriented in XZ- and XY-directions.

For all specimens, a 3PBT with a support span of 100 mm is conducted until failure, which is defined as crack propagation over the entire specimen height, accompanied by a sudden load drop. The different orientations of the layers determine the specimen names, with the *x*-axis of the applied coordinate system consistently aligned with the longitudinal axis of the specimen (see Figure 3). The *x*–*y* plane spans the surface for optical measurements.

The experimental testing arrangement is divided into two series. Series I comprises specimens without CJ in three different layer orientations: XZ, XY, and YZ (see Figure 3a). Series II focuses on specimens with CJ oriented in XZ- and XY-directions (Figure 3b).

All tests are performed displacement-controlled at a rate of 400 μm/min. As displacement is induced, the applied machine force is continuously monitored at the midpoint of the specimen. In addition, the displacement and strain fields on the specimen’s front side are continuously measured (see Section 2.4).

Table 2 presents an overview of the conducted tests and the number of samples for each series. External conditions led to a varying specimen age between the two series. Additionally, two specimens produced under the same conditions as the CJ specimens but without pauses in printing are available for testing. These specimens serve as a valuable reference for Series II without CJ, providing additional insight despite the limited sample size.

### 2.4. Optical Measurements

**Figure 4:**
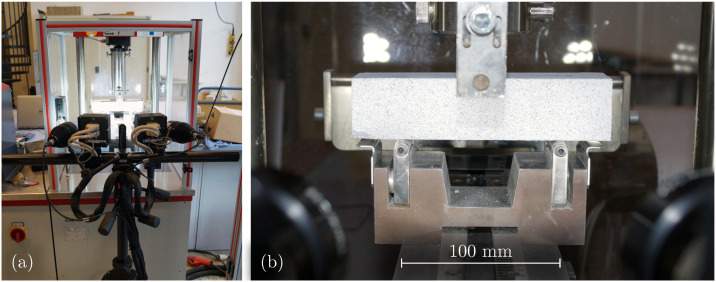
(**a**) Three-point bending test setup with ARAMIS 4M optical stereo camera system. (**b**) Specimen with sprayed stochastic grey value pattern on the surface.

In order to measure deformations on the specimen’s surface, the contactless optical stereo camera system ARAMIS 4M manufactured by GOM GmbH, Braunschweig, Germany (now Carl Zeiss Industrial Quality Solutions GmbH, Oberkochen, Germany; Figure 4a) is used. The system consists of two cameras and one sensor that employs DIC measurement methods. By recognizing specific image areas in both cameras, the system can measure 3D displacements and surface strains [41]. To obtain a suitable grey value image without influencing the mechanical behavior, the specimens are sprayed with a thin layer of paint (see Figure 4b).

The measurement accuracy for the specified measurement volume is 0.01 mm. Images are captured with a sampling frequency of $f_{s}=2\;Hz$. Additionally, the system features a ring memory configured to store the last 250 images at an increased frequency of $f_{s}=50\;Hz$.

## 3. Results

### 3.1. Three-Point Bending Test Series I

#### 3.1.1. Failure Mechanisms

**Figure 5:**
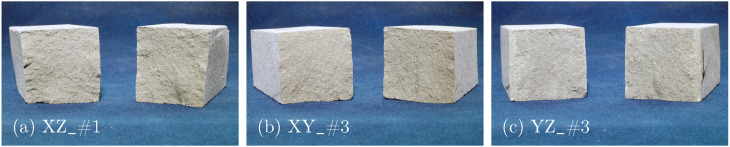
Fracture surfaces of 3PBT for Series I: specimens with layers oriented in (**a**) XZ-, (**b**) XY- and (**c**) YZ-direction.

As expected, the failure mechanism of the unreinforced specimens in 3PBT is characterized by a discrete crack at the beam center. The fracture surface appears homogeneous, reflecting the high printing quality (see Figure 5).

The central crack leading to failure initiates at a corresponding crack force $F_{\mathrm{cr}}$. To enable an accurate comparison across all specimens, the crack force is adjusted to a normalized force, $F_{\mathrm{cr},\mathrm{norm}}$, based on a standardized geometry. This adjustment accounts for dimensional variations in the actual cut specimens. The normalized geometry equals the target cross-sectional dimensions and the support span. Assuming identical flexural tensile strength $f_{t}$ for both the normalized specimen and each tested specimen, $F_{\mathrm{cr},\mathrm{norm}}$ is calculated by:

(1) $$F_{\mathrm{cr},\mathrm{norm}}=F_{\mathrm{cr}}\;\cdot \;\frac{b_{\mathrm{norm}}\;\cdot \;h_{\mathrm{norm}}^{2}}{b\;\cdot \;h^{2}}\;,$$

with the following definitions:

- *b* and *h*: measured width and height, respectively;
- $b_{\mathrm{norm}}=40\;\mathrm{mm}$: normalized width;
- $h_{\mathrm{norm}}=40\;\mathrm{mm}$: normalized height;
- $L_{\mathrm{norm}}=100\;\mathrm{mm}$: normalized length.

**Figure 6:**
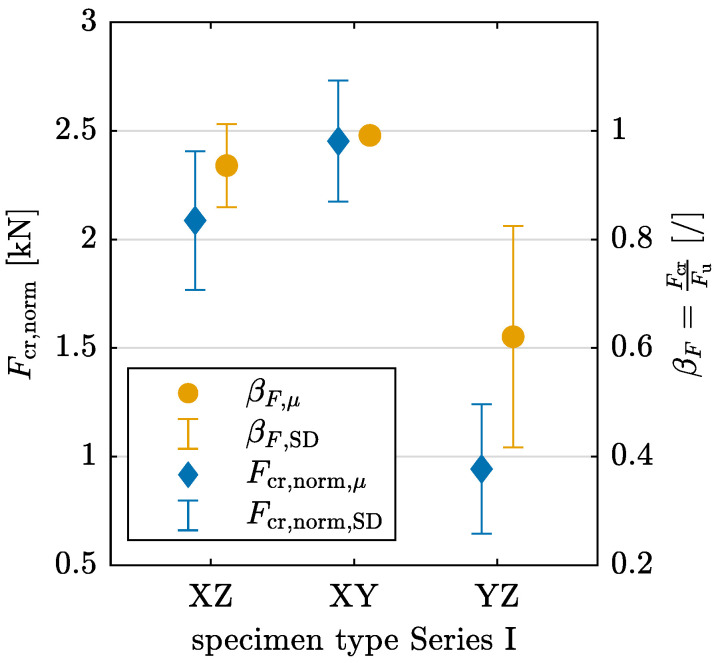
Normalized crack force $F_{\mathrm{cr},\mathrm{norm}}$ and ratio $\beta_{F}=\frac{F_{\mathrm{cr}}}{F_{u}}$ of crack force $F_{\mathrm{cr}}$ and failure load $F_{u}$ derived from 3PBT for layer orientations XZ, XY, and YZ in Series I with mean value μ and standard deviation SD.

Figure 6 visualizes the mean values and standard deviations of $F_{\mathrm{cr},\mathrm{norm}}$ for each specimen orientation as well as the crack force in relation to the ultimate failure load $F_{u}$. For YZ specimens, the crack initiates at a notably lower load level compared with the other orientations. Additionally, for the XZ and XY orientations, the ratio $\beta_{F}$ has values close to 1, indicating that crack initiation occurs just before reaching the failure load. In contrast, for YZ specimens, the crack develops over a broader load range, demonstrating a distinct mechanical behavior for this orientation.

An analysis of variance (ANOVA) is conducted to further evaluate the statistical significance of the obtained results for $F_{\mathrm{cr},\mathrm{norm}}$ and $\beta_{F}$. A statistically significant difference in the mean values for the different interlayer orientations is defined for a *p*-value p<0.05, corresponding to a confidence interval of 95%. The one-way ANOVA yields *p*-values of p=0.0227 for $F_{\mathrm{cr},\mathrm{norm}}$ and p=0.0020 for $\beta_{F}$, supporting the alternative hypothesis of a significant difference between the interlayer orientations. A multiple comparison test, based on Tukey’s honestly significant difference procedure, confirms a significant difference for the YZ orientation compared with the other two interlayer orientations for both dependent variables, $F_{\mathrm{cr},\mathrm{norm}}$ and $\beta_{F}$.

**Figure 7:**
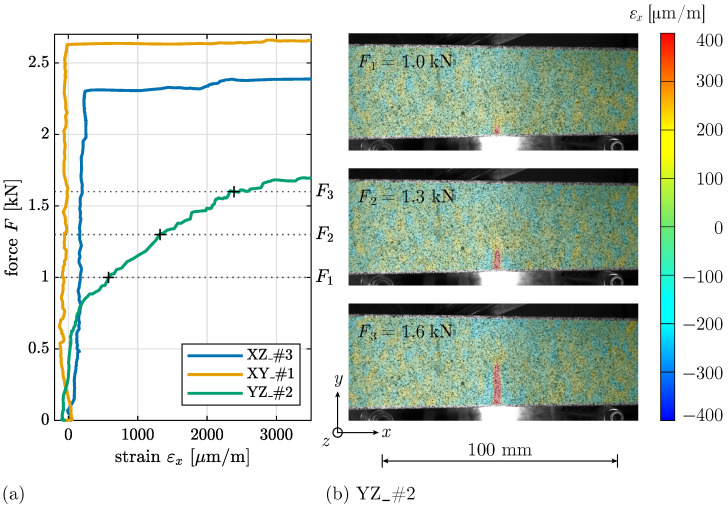
(**a**) Evolution of machine force *F* over strain $\varepsilon_{x}$ in failure crack for specimens XZ_#3, XY_#1, and YZ_#2. (**b**) Strain field $\varepsilon_{x}$ with crack evolution for specific load levels $F_{1}$ to $F_{3}$ for specimen YZ_#2.

The distinct mechanical behavior of YZ specimens is further illustrated in Figure 7a, which presents the machine force *F* in relation to the strain $\varepsilon_{x}$ at the location of the central crack, obtained at the bottom edge of a specimen from each layer orientation group using optical measurement data. The gradient of the curves for XZ and XY orientations is nearly vertical, shifting to horizontal, showing that strain increases only shortly before the failure load is reached. In contrast, for the YZ orientation, force and strain increase continuously. Figure 7b illustrates the gradual crack development in the YZ orientation through the strain field $\varepsilon_{x}$ at different load levels $F_{1}$ to $F_{3}$.

**Figure 8:**
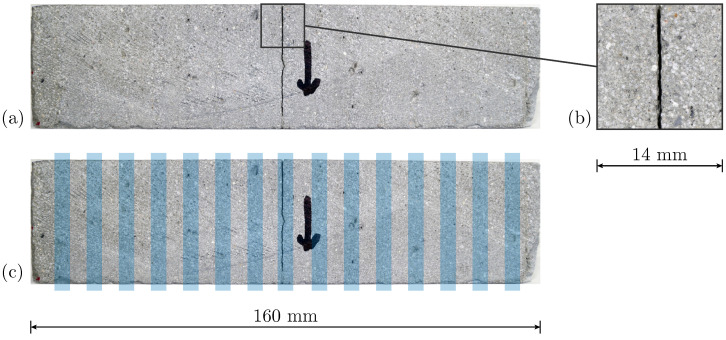
Specimen YZ_#3 with arrow mark indicating printing direction: (**a**) surface of broken specimen with (**b**) detail of crack area and (**c**) highlighted interlayer regions.

The surface illustration of a YZ specimen in Figure 8a reveals distinct interlayer regions, further highlighted in Figure 8c. Across all YZ specimens, cracks consistently initiate and propagate within these interlayer regions, where material properties are expected to be comparatively weaker, as documented in prior studies [15,20].

#### 3.1.2. Material Properties

The observed failure mechanisms reflect load-bearing behavior influenced by layer orientation, suggesting an inhomogeneous material distribution. To facilitate further comparison of the material’s anisotropic characteristics, subsequent analyses of displacements and strain values assume homogeneous, linear-elastic material behavior. The Young’s modulus can be determined from 3PBT using linear beam theory:

(2) $$E=\frac{\Delta F\;\cdot \;L^{3}}{\Delta u\;\cdot \;48I}\;,$$

with the following definitions:

- $I=\frac{b\;\cdot \;h^{3}}{12}$ is the moment of inertia;
- *L* is the support span;
- ΔF and Δu are the incremental values for force and displacement, respectively.

The displacement is determined from the optical measurement data at the load application point on the top edge of the beam center. ΔF and Δu are calculated through a linear fit of each specimen’s force-displacement curve, a reasonable approximation given the failure occurring in the tensile zone. The exact cross-sectional dimensions *b* and *h* are measured individually for each specimen.

With the recorded ultimate load *F*, the flexural tensile strength $f_{t}$ results in

(3) $$f_{t}=\frac{F\;\cdot \;\frac{L}{4}}{\frac{b\;\cdot \;h^{2}}{6}}=\frac{3\;\cdot \;FL}{2\;\cdot \;bh^{2}}\;.$$

**Figure 9:**
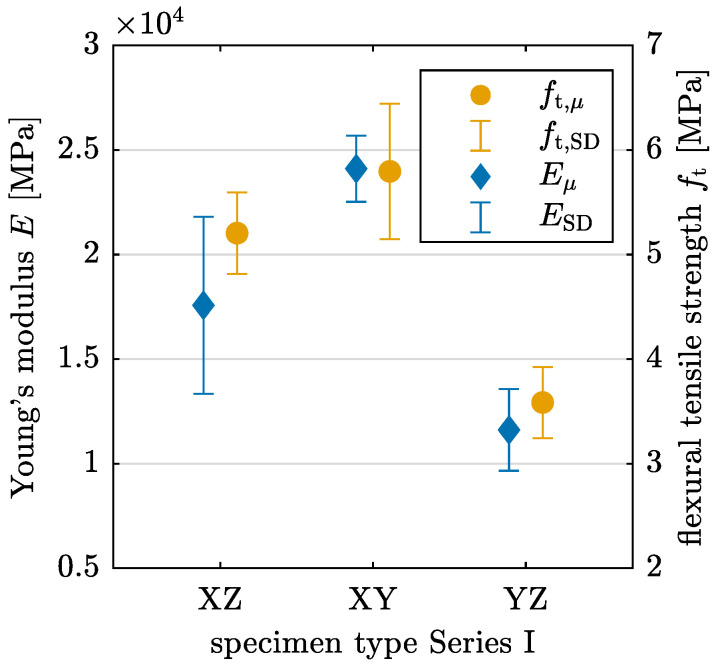
Young’s modulus *E* and flexural tensile strength $f_{t}$ derived from 3PBT for layer orientations XZ, XY, and YZ in Series I with mean value μ and standard deviation SD.

The results for both Young’s modulus and flexural tensile strength are presented in Table 3 and visualized in Figure 9. The values align with the observations from crack development, indicating that the mean value of *E* is up to 51.8% lower for YZ-oriented specimens compared with the other orientations. Specimens with XY-oriented layers yield the highest mean value for *E*. A similar trend is noted for flexural tensile strength, following the tendencies observed for Young’s modulus across the different specimen orientations.

The anisotropy in Young’s modulus and flexural tensile strength is statistically verified for the YZ orientation, with *p*-values of p=0.0051 for *E* and p=0.0033 for $f_{t}$ determined by an ANOVA. A significant difference in the mean values of *E* is observed between XY and YZ orientations. For $f_{t}$, the YZ orientation exhibits a statistically significant difference compared with the other two orientations.

### 3.2. Three-Point Bending Test Series II

#### 3.2.1. Failure Mechanisms

**Figure 10:**
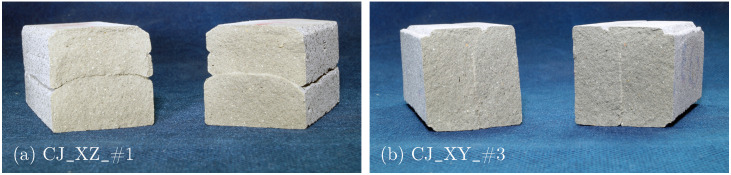
Fracture surfaces of 3PBT for Series II: specimens with CJ oriented in (**a**) XZ- and (**b**) XY-direction.

The failure mechanisms for Series II are fundamentally similar to those in Series I, characterized by brittle failure with a dominant crack initiated when the tensile stress at the bottom of the specimen exceeds its tensile strength. In the fracture surface, the CJ are clearly distinguishable in both horizontal and vertical directions as shown in Figure 10a and Figure 10b, respectively.

**Figure 11:**
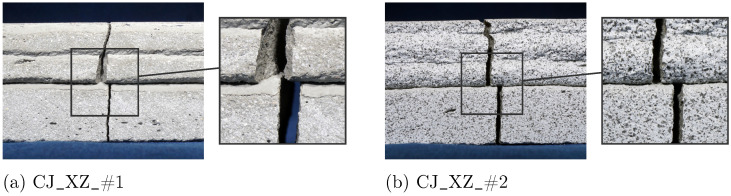
Detail of crack path resulting from 3PBT for Series II for specimen (**a**) CJ_XZ_#1 and (**b**) CJ_XZ_#2.

Interestingly, for specimens with CJ, an offset in the propagated crack can be observed precisely at the location of the CJ, as shown in Figure 11. The CJ act as an interlayer with altered bonding characteristics and therefore influence the development of the crack path.

**Figure 12:**
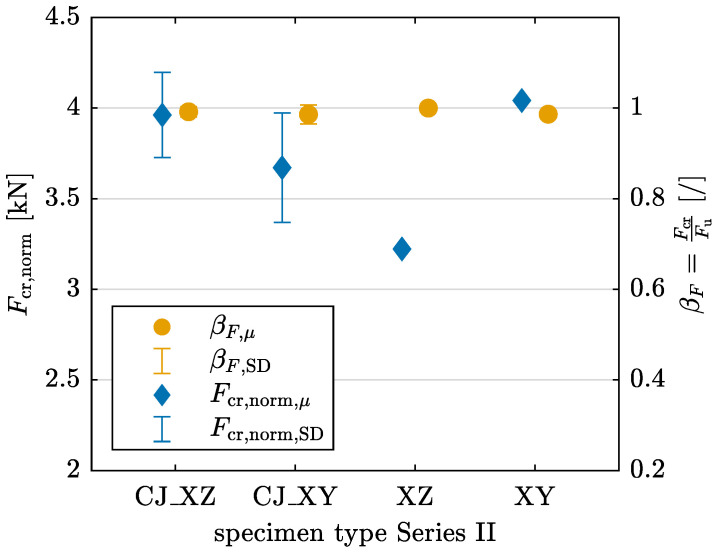
Normalized crack force $F_{\mathrm{cr},\mathrm{norm}}$ and ratio $\beta_{F}=\frac{F_{\mathrm{cr}}}{F_{u}}$ of crack force $F_{\mathrm{cr}}$ and failure load $F_{u}$ derived from 3PBT for specimens with and without CJ and layer orientations XZ and XY in Series II with mean value μ and standard deviation SD.

As with Series I, a normalized crack force $F_{\mathrm{cr},\mathrm{norm}}$ (see Equation (1)) and the ratio $\beta_{F}$ are calculated. For $\beta_{F}$, it can clearly be seen in Figure 12 that all cracks develop suddenly, as the ratio is nearly 1 for all specimens. While $F_{\mathrm{cr},\mathrm{norm}}$ is lower for the XZ specimen without CJ (see Figure 12), this difference should be interpreted cautiously, as only limited data from a single specimen are available.

The ANOVA yields *p*-values of p=0.1515 for $F_{\mathrm{cr},\mathrm{norm}}$ and p=0.8721 for $\beta_{F}$. Consequently, the null hypothesis is accepted, indicating no significant variation in either $F_{\mathrm{cr},\mathrm{norm}}$ or $\beta_{F}$ for Series II.

#### 3.2.2. Material Properties

**Figure 13:**
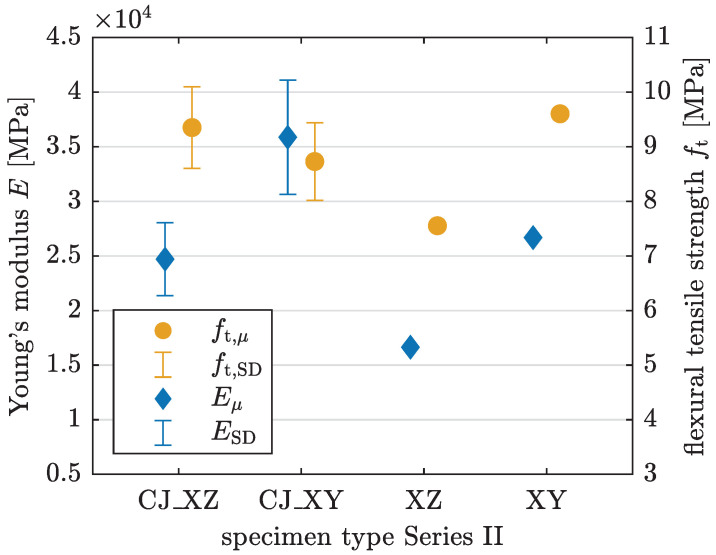
Young’s modulus *E* and flexural tensile strength $f_{t}$ derived from 3PBT for specimens with and without CJ and layer orientations XZ and XY in Series II with mean value μ and standard deviation SD.

The homogenized material properties are determined in the same manner as for Series I; see Equations (2) and (3). Table 4 and Figure 13 present the results. Since there is only one specimen without CJ tested for each layer orientation, the corresponding standard deviations and coefficients of variation equal zero.

Flexural tensile strength yields values in a similar range across most specimens in Series II, reaching up to 9.6 MPa. The ANOVA for $f_{t}$ indicates that the difference between group means is not statistically significant (p=0.2441>0.05). The Young’s modulus for the XZ-oriented specimen, at 16,637 MPa, is the lowest among the samples (see Table 4). The *p*-value from a one-way ANOVA for *E* (p=0.0364) indicates a significant difference, with the multiple comparison test confirming a significant difference only between the mean values of the CJ_XY and XZ orientations. CJ_XY specimens achieve the highest value for *E*, while specimens without CJ have comparatively lower *E* values for the corresponding interlayer orientations. As only a single specimen without CJ is available for each orientation, a definite cause for this result cannot be established. Further investigations are necessary.

## 4. Discussion

The experimental investigation of 3PBT on specimens with various interlayer orientations in Series I, along with the inclusion of CJ in Series II, enables a differentiated analysis of material behavior. The results from Series I confirm that the layered deposition process induces anisotropic characteristics in the material. This anisotropy is evident in the pronounced dependency of strength and stiffness properties on layer orientation. Statistically significant reductions in Young’s modulus and flexural tensile strength are observed under homogenized material assumptions for the YZ orientation, while specimens with XY-oriented layers exhibit slightly higher material parameters than those with XZ-oriented layers. These trends are consistent with findings reported in [37], where different printing patterns and layer orientations were analyzed in 3PBT. Despite the high printing quality achieved in the present study, orientation-dependent effects remain evident.

The analysis reveals distinct failure mechanism patterns that align with the obtained results for material parameters. Cracks develop suddenly in all configurations except for YZ-oriented specimens, as indicated by the normalized crack force and the ratio $\beta_{F}$ (see Figure 6 and Figure 12). For YZ-oriented specimens, cracks form along the interlayer region, similar to findings reported in [27]. In contrast, for Series II, an offset in crack path is observed in CJ_XZ specimens (Figure 11), a behavior that aligns with observations in [28], where horizontally deviating cracks at CJ were linked to interlayer interval time. CJ introduce a designated weak point that influences crack propagation.

Regarding stiffness, similar trends to Series I are observed in Series II. Specimens with XY-oriented layers show a 45% to 60% higher mean Young’s modulus compared with those with XZ orientation, observable across both CJ and non-CJ specimens. In contrast, the variance in flexural tensile strength exhibits less statistical significance.

In a direct comparison of the absolute values of material parameters, the values for *E*, $f_{t}$, and $F_{\mathrm{cr}},\mathrm{norm}$ in Series II are notably higher than those in Series I. These results are primarily linked to differences in testing age, as material strength increases over time. However, the degree of increase exceeds expectations. While the Fib Model Code for Concrete Structures comprises functions to account for time-dependent increases in strength and stiffness [42], the observed results suggest that conventional approaches may not fully apply to 3DCP. Shorter interlayer interval times likely enhance the interlayer bond by reducing water evaporation, leading to higher strength, as discussed in [21]. The interlayer interval time in this study differs between the two series by 44 s due to a distinct printed geometry (see Section 2.1). A variation of less than 1 min is initially not expected to significantly impact the interlayer bond. Nevertheless, this factor, along with sawing at a later stage, which potentially causes less disruption of the microstructure in Series II, might influence the results. Environmental factors, such as temperature, humidity, and curing, generally play a significant role, as demonstrated in [21,43]. However, since sample preparation was conducted under identical environmental conditions, these factors are not considered decisive in the present work.

When comparing CJ specimens with the reference specimens without CJ in Series II, the commonly reported inferior mechanical performance due to CJ does not manifest clearly in this study. Nevertheless, the available data are limited, and further research is required to draw final conclusions.

## 5. Conclusions

The present study provides valuable insights into the mechanical behavior of 3DCP, particularly highlighting the effects of layer orientation and CJ on the structural performance. The experimental setup employs displacement-controlled 3PBT with a support span of 100 mm and includes two series containing hardened printed concrete specimens with and without CJ at the mid-plane, each with varying layer orientations. Specimens are fabricated following the interlaboratory study guidelines established by RILEM TC 304-ADC [35]. The application of DIC measurement methods allows for both a qualitative analysis of failure mechanisms and a quantitative investigation of strength, stiffness, and force development over strain.

The results confirm a strong dependency on layer orientation from a structural perspective, demonstrating the anisotropic behavior of printed concrete, independent of the presence of CJ. Key material parameters, including Young’s modulus and flexural tensile strength, vary significantly based on layer orientation, with YZ-oriented specimens exhibiting notably weaker mechanical properties.

Failure mechanisms and crack development reveal the weakest performance in YZ-oriented specimens, where cracks initiate gradually and at a lower relative load level compared with the other orientations, which exhibit sudden crack propagation. The crack development is concentrated in the interlayer regions, underscoring weak interlayer adhesion. CJ noticeably affect crack development, causing deviations in the crack path precisely at the CJ location. These results are relevant in practical applications, where CJ formed during the printing process can act as planes of weaknesses, potentially compromising structural integrity.

Characteristic parameters for CJ specimens are generally higher than those for specimens without CJ, most probably due to the increased age of the specimens at the time of testing. Further studies testing both CJ and non-CJ specimens at comparable, increased ages would provide an additional perspective, since most studies are focused on the standard testing age of 28 days. It remains unclear whether the influence of CJ may decrease over time. Extended research is necessary for complete interpretation of these results.

Conventional design strategies for concrete assume isotropic material behavior. Consequently, understanding the anisotropic characteristics introduced by the 3DCP manufacturing process is crucial for enhancing material properties through optimized printing parameters and curing techniques or for developing structural designs that align with the altered material properties.

This study demonstrates that anisotropies in printed concrete are detectable at a structural level. Directional dependencies significantly influence load-bearing behavior, making it essential to identify and comprehend these weaknesses to ensure structural integrity. As a result, predictive computational models must explicitly account for the anisotropic material behavior and potential weaknesses introduced by CJ. The findings from this study contribute to a fundamental understanding of the structural performance of hardened printed concrete and provide a comprehensive foundation for further research, including numerical modeling.

## Figures and Tables

**Table 1:** Mortar composition developed by Rasehorn, with material quantities specified for 1 m^3^ of printable mortar (calculated density of 2.274 kg m^−3^, extruded density of ≈2.167 kg m^−3^).

Component	Manufacturer	Density [kg m^−3^]	Mass [kg]	Specification
CEM I 52.5 R	CEMEX (Rüdersdorf, Germany)	3107	604.0	
Water	-	1000	242.0	water/cement ratio of 0.40
Sand 0/1	SAND-SCHULZ (Berlin, Germany)	2656	967.0	
FA	BauMineral (Herten, Germany)	2325	193.0	
LS	CEMEX	2720	145.0	
MS	DuraPact (Haan, Germany)	2312	97.0	
Fibers	DBF (Sangerhausen, Germany)	2727	4.8	
SP	Sika (Stuttgart, Germany)	1060	14.5	2.4% by weight of cement
Deaerator	Sika	990	6.8	1.1% by weight of cement

**Table 2:** Overview of specimens for 3PBT.

Test	Series	Specimen Age	Layer Orientation	Number of Specimens
Three-point bending test (3PBT)	Series I	34 days	XZ	3
			XY	3
			YZ	3
	Series II	148 days	CJ_XZ	3
			CJ_XY	4
			XZ	1
			XY	1

**Table 3:** Young’s modulus *E* and flexural tensile strength $f_{t}$ derived from 3PBT for layer orientations XZ, XY, and YZ in Series I with mean value μ, standard deviation SD, and coefficient of variation CV.

Layer Orientation	Young’s Modulus *E*				Flexural Tensile Strength $f_{t}$		
	μ [MPa]	SD [MPa]	CV [%]		μ [MPa]	SD [MPa]	CV [%]
XZ	17573	4239	24.1		5.20	0.39	7.5
XY	24107	1585	6.6		5.80	0.65	11.2
YZ	11613	1954	16.8		3.59	0.34	9.5

**Table 4:** Young’s modulus *E* and flexural tensile strength $f_{t}$ derived from 3PBT for specimens with and without CJ and layer orientations XZ and XY in Series II with mean value μ, standard deviation SD, and coefficient of variation CV.

Layer Orientation	Young’s Modulus *E*				Flexural Tensile Strength $f_{t}$		
	μ [MPa]	SD [MPa]	CV [%]		μ [MPa]	SD [MPa]	CV [%]
CJ_XZ	24702	3339	13.5		9.35	0.75	8.0
CJ_XY	35867	5238	14.6		8.73	0.71	8.1
XZ	16637	0	0.0		7.55	0.00	0.0
XY	26677	0	0.0		9.60	0.00	0.0

## Data Availability

The raw data supporting the conclusions of this article will be made available by the authors on request due to its inclusion in an ongoing study.

## Abbreviations

The following abbreviations are used in this manuscript:

3DCP	3D concrete printing
3PBT	Three-point bending test
ANOVA	Analysis of variance
CJ	Cold joints
DIC	Digital image correlation
FA	Fly ash
LS	Limestone powder
MS	Microsilica
SP	Superplasticizer
